# Why do startups fail? A core competency deficit model

**DOI:** 10.3389/fpsyg.2024.1299135

**Published:** 2024-02-08

**Authors:** Edit Szathmári, Zoltán Varga, Attila Molnár, Gergely Németh, Zsolt Péter Szabó, Orhidea Edith Kiss

**Affiliations:** ^1^Doctoral School of Psychology, ELTE Eötvös Loránd University, Budapest, Hungary; ^2^Institute of Psychology, ELTE Eötvös Loránd University, Budapest, Hungary; ^3^Independent Researcher, Budapest, Hungary; ^4^TeamINSIGHT Labs Ltd., Budapest, Hungary; ^5^Institute of Management and Finance, Budapest Metropolitan University, Budapest, Hungary; ^6^Corporate Values Kft., Budapest, Hungary; ^7^Institute of Strategy and Management, Corvinus University of Budapest, Budapest, Hungary; ^8^Institute of Psychology, University of Pécs, Pécs, Hungary

**Keywords:** startup failures, core competencies, competency deficits, information-seeking, customer service orientation

## Abstract

A growing body of work aims to explore the reasons behind startup failures. However, there is a need for integrative approaches organized around conceptual frameworks to avoid fragmented and perplexing knowledge about these reasons. To our knowledge, no previous research has systematically investigated the role of competency deficits in startup failures, a crucial element of these failures. In our study, we adapted Spencer’s behavioral competence model specifically for startups to identify the competencies within startup teams that, according to their Chief Executive Officers, contributed to their downfall. Three coders meticulously analyzed 50 online accounts of startup failures using a modified Critical Incident Technique. This analysis revealed two prominent competency deficits as pivotal determinants of these startups’ outcomes: information-seeking and customer service orientation. Additionally, deficits in technical expertise, analytical thinking, and flexibility emerged as significant factors contributing to these failures. The competency deficits identified in this study offer focal points for evaluating and enhancing startup teams, thereby helping to prevent failure.

## 1 Introduction

A growing body of work has sought to explore the predictors of startup successes and failures. Despite the lack of consensus on what constitutes a startup (Knight et al., 2020), definitions typically include elements such as equity ownership, autonomy in strategic decision-making, and entitativity (for a review, see Knight et al., 2020). In other words, a startup is a team in which team members have a financial interest, the team possesses decision-making authority and agency, and the team is a social entity with distinct boundaries. Furthermore, aligning with Blank (2013), Bruyat and Julien (2001), and Kaczam et al. (2022), we define a startup as the initial stage of an entrepreneurial venture that is still in search of a repeatable and scalable business model with limited financial resources. This phase is often funded by external investors or the founding team members (Skala, 2019; Bianco et al., 2022). The Chief Executive Officer (CEO) of the startup, typically one of the founders at this stage, faces specific roles and challenges distinct from those in more established organizations (Picken, 2017; Wang et al., 2022).

There is a constant need to understand startup success and failure, given that various statistics indicate that the failure rates are around 90% (Marmer et al., 2012a; Giardino et al., 2014; Startup Genome LLC, 2018, 2022). Additionally, Griffin (2017) points out that the majority of startups fail in their very early stages of operation, emphasizing the critical need for a deeper understanding of the reasons behind these failures. In this context, we begin by briefly discussing and reviewing existing research on startup failures.

### 1.1 Predictors of startup failures

There is a consensus in the literature that the failure of a startup is rarely attributable to a single factor (Cantamessa et al., 2018). Reasons for startup failures can be categorized into two main groups: internal and external factors (CB Insights, 2021; Öndas and Akpinar, 2021). However, the distinction between these categories is not always straightforward. For instance, Öndas and Akpinar (2021) classify a lack of sufficient customers to cover recurring expenses as an external factor. Yet, this issue can also be connected to internal factors, such as inadequate business plans and strategies.

Empirical studies often examine one or a few predictors of startup failures, or they identify multiple factors without proposing a clear theoretical framework to systematize these underlying reasons (Littunen et al., 1998; Lussier and Pfeifer, 2001; Rogoff et al., 2004; Cardon et al., 2011; Giardino et al., 2014; Khelil, 2016; Krishna et al., 2016; Bednár and Tarišková, 2017; Mikle, 2020; Santisteban et al., 2022; Joseph et al., 2023). For instance, Bednár and Tarišková (2017) list 13 different factors, including lack of money, cost issues, and lack of investors, among others. Their categorization of these factors into broader categories is primarily based on empirical findings, specifically frequency data extracted from founders’ statements, and does not rely on a strong theoretical foundation. They argue that the most common causes of startup failures are financial issues, market gaps, and team shortcomings. Giardino et al. (2014) reached similar conclusions, identifying four overarching dimensions (the team, the product, the business, and the market) as key contributors to early-stage software startup failures, a finding that echoes earlier research (Macmillan et al., 1987; Öndas and Akpinar, 2021).

In their review of predictors of startup failure, Akter and Iqbal (2020) focused on six types of failure factors. They argued that startup failures result from a combination of organizational factors (e.g., lack of strategy), product factors (e.g., user-unfriendly product), human factors (e.g., lack of commitment), financial factors (e.g., lack of cash and financing possibilities), market factors (e.g., strong competition), and ecosystem factors (e.g., legal challenges).

Similarly, Triebel et al. (2018) proposed that the five main reasons for failures are capital procurement, technological concept/implementation risks, market opportunities/market hurdles, personal or team-specific reasons, and other reasons. They emphasize the significance of team-internal reasons and argue that factors such as market opportunities/market hurdles can also be considered as team-internal reasons (Triebel et al., 2018).

Gruber et al. (2008) focused on more specific reasons, finding that novice entrepreneurs identify fewer market opportunities for their technologies before their first market entry compared to serial entrepreneurs, making them more susceptible to failure. This could be conceptualized as an information-seeking problem. A subsequent study by Gruber et al. (2013) suggested that the identification of market opportunities is also influenced by the founding team’s industry experience and external knowledge-sourcing relationships. More diverse industry experiences and external knowledge-sourcing relationships increase the variety of market opportunities identified by the startup team, which, in turn, leads to a higher likelihood of subsequent diversification.

While the literature reviewed above has provided significant insights into the causes of startup failures, there is a pressing need for more integrated approaches centered around conceptual frameworks to prevent the accumulation of fragmented (in terms of failure reason categories) and somewhat confusing (in distinction between these categories) knowledge about startup failures. The work by Cantamessa et al. (2018) and Öndas and Akpinar (2021) exemplify such integrated approaches.

Utilizing a startup-specific adaptation of the SHELL model, a well-known approach in the aviation sector for describing accident causes, these authors present a taxonomy of the causes behind high-tech startup failures and offer a toolkit for conducting a multi-dimensional analysis of these failures. In Cantamessa et al.’s (2018) adaptation of the SHELL model, the business model is analogous to the software (S), the product to the hardware (H), the startup’s environment to the accident environment (E), the organization to the central liveware (L), and the customer/user to the liveware (L).

In the modified SHELL model by Öndas and Akpinar (2021), managerial issues align with the software component, product-related problems with the hardware component, financial challenges with the environmental component, and market-related problems with the liveware component. Cantamessa et al. (2018) conclude that the absence of a structured business development strategy is a pivotal predictor of startup failure, while Öndas and Akpinar provide recommendations to address concerns related to each component of their model.

However, it is important to note that while the startup-specific SHELL model offers valuable insights, it still has limitations in providing practical suggestions for future startups due to its encompassing a wide range of both internal and external predictors.

Klotz et al. (2014) emphasize the importance of investigating new venture teams, rather than focusing solely on individual entrepreneurs, given that most ventures are initiated by teams. Within the New Venture Team (NVT) Input-Mediators-Outcome (IMO) framework, they propose that the connections between inputs (e.g., prior experience, personality) and outcomes closely tied to the success or failure of the startup (e.g., profitability, sales growth) are mediated by team processes (e.g., team conflict) and emergent states (e.g., cohesion).

Industry experts also highlight the paramount importance of forming a strong team, more so than other investment factors, in early-stage startups (Baum et al., 2000; Honoré, 2022). In a longitudinal study, Van Gelderen et al. (2006) found that the lack of knowledge, skills, education, and experience to effectively operate a team, coupled with push motivations and high ambitions, can contribute to failure among nascent entrepreneurs.

Through their systematic literature review, Santisteban and Mauricio (2017) identified ten team-related factors that influence the success of IT startups. Among these factors, two are related to competence: the leadership of the entrepreneur and the technological/ business capabilities of the founding team. Given this, the capabilities and competencies of the team, as internal human factors, play a significant role in failure. We now shift our focus to core competencies as a potential framework to understand and organize the driving forces behind these elements of failure.

### 1.2 Applying Spencer’s competency model in the startup context

Core competencies can be defined as a ‘combination of motives, traits, self-concepts, attitudes or values, content knowledge or cognitive behavior skills’ (Spencer and Spencer, 1993, p. 4). To evaluate the competencies of startup teams as determinants of their failure, we needed a well-established general competency model as a foundation, which we then adapted to suit the startup context. While research interest in the distinct competencies of startup teams is growing (e.g., Santisteban and Mauricio, 2017; Seppänen et al., 2017; Assyne and Wiafe, 2019), a comprehensive startup-specific competency model has yet to be developed.

A startup-specific core competency model would posit that the presence of core competencies distinguishes successful from unsuccessful performers. Given their size, these units can be viewed as ‘small teams,’ where the composition of competencies is considered complementary (Katzenbach and Smith, 1993). This implies that the group itself, rather than individual team members, should possess these core competencies at an aggregate level.

Considering its expansive application history (Sanghi, 2007), we chose Spencer’s competency model as a foundation (Spencer and Spencer, 1993) and modified it to create a startup-specific core competency model. Spencer’s model contains 20 competencies distributed across six clusters: (1) achievement and action (competencies: achievement orientation; concern for order, quality, and accuracy; initiative; information-seeking); (2) helping and human service (competencies: interpersonal understanding; customer service orientation); (3) impact and influence (competencies: impact and influence; organizational awareness; relationship building); (4) managerial (competencies: developing others; directiveness: assertiveness and use of positional power; teamwork and cooperation; team leadership); (5) cognitive (competencies: analytical thinking; conceptual thinking; technical/professional/managerial expertise); and (6) personal effectiveness (competencies: self-control; self-confidence; flexibility; organizational commitment).

Spencer’s model offers several advantages: (1) it encompasses relevant competencies for startups, (2) it maintains a manageable number of competencies organized into clusters; and (3) its application has a proven track record (Sanghi, 2007; Megahed, 2018). Both indirect and direct evidence support the role of these competencies and competency clusters in startup failures. For instance, Gruber et al. (2008, 2013) linked startup failures to insufficient information-seeking and market orientation (see Spencer’s information-seeking and customer service orientation competencies), Santisteban and Mauricio (2017) to inadequate managerial competencies (see Spencer’s managerial cluster), and Assyne and Wiafe (2019) to deficiencies in cognitive competencies (see also Carpentier and Suret, 2015; Seppänen et al., 2017) (see Spencer’s cognitive cluster), while Carpentier and Suret (2015) pointed to a lack of personal effectiveness in entrepreneurs (see also Klotz et al., 2014) (see Spencer’s personal effectiveness cluster).

### 1.3 Aims of the study

In the current study, we analyzed the narrative accounts, referred to as post-mortems, provided by leaders of 50 failed startup teams. Our goal was to explore and identify core competency deficits within these teams. Given our objective, we chose a qualitative approach. Specifically, we employed the Critical Incident Technique (Flanagan, 1954), the details of which are elaborated in the Methods section.

To the best of our knowledge, no previous research has systematically investigated the role of core competencies in the context of startup failures. Initially, each competency cluster seemed relevant, with both direct and indirect evidence linking them to startup failures, as discussed in our preceding review. Consequently, we formulated open-ended research questions rather than specific hypotheses. Our research questions were: “Is it possible to categorize the absence of specific core competencies as contributing factors to startup failures?” (RQ1) and “Which core competencies are most frequently mentioned in the narrative accounts of startup leaders as explanations for failure?” (RQ2).

## 2 Method

### 2.1 Sample characteristics: narrative accounts of startup failures

Following the recommended sample size for Critical Incident Technique studies (Flanagan, 1954; Bott and Tourish, 2016), we analyzed a total of 50 instances of startup failures. From a qualitative narrative analysis perspective, this can be considered a rather large sample (Braun and Clarke, 2021). We opted for this sample size in line with our broader interest in startups, not exclusively limited to the most frequently investigated technological startups (e.g., Nummela et al., 2016; Bajwa et al., 2017; Santisteban and Mauricio, 2017; Seppänen et al., 2017; Assyne and Wiafe, 2019; Gbadegeshin et al., 2022). This approach allowed us to assemble a diverse collection of startup narratives.

The failure accounts, referred to as post-mortem testimonials, were sourced from the internet in 2018 using search keyword combinations, specifically ‘startup + fail story’ and ‘startup + post-mortem’. The inclusion criteria for these narratives were: (1) the post-mortem testimonial was a firsthand account by a startup CEO regarding their own experience of failure, (2) the testimonial provided more than a brief mention of the startup’s failure – it detailed the process and included the CEO’s perspective, (3) the date of the failure was between 2014 and 2017, comprising 47 testimonials. However, we also included the first three hits outside of this time interval to reach the recommended sample size. Twenty-four stories were sourced from medium.com, ten from various startup-specific online magazines and blogs (eight stories from different sources and two from techcrunch.com), and sixteen from the startups’ official websites. Among these narratives, forty-seven stories were composed in English, and three in Hungarian.

**Figure 1:**
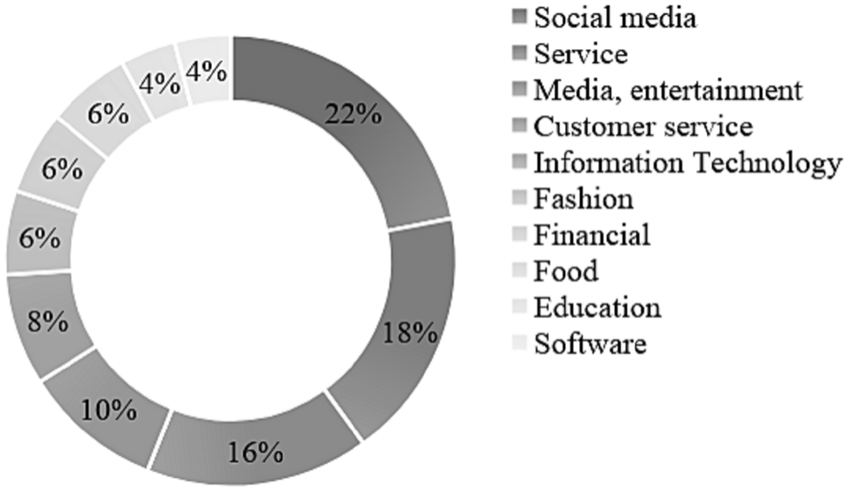
Distribution of startups by industry in the sample.

In the majority of the cases, the CEO was male (88%), with one instance where the CEO position was jointly held by a male and a female, and five cases (10%) featuring a female CEO. The startups originated from 23 different countries across various geographical regions: 20% in California; 28% in other US and Canadian states; 26% in Europe; 22% in the Asia-Pacific region; and 2% in Latin-America. In one case, the region of operation could not be determined. Overall, 56% of the sample operated in the “business-to-customer” (B2C) domain, 34% in the “business-to-business” (B2B) domain, and 10% targeted both businesses and customers with their products/services. Two-thirds of the ventures in the sample operated within social media (22%), service (18%), media-entertainment (16%), and customer service (10%) industries. Among the startups in the sample, 47 experienced failure between 2014 and 2017, while the remaining three ceased operations in 2013, 2012, and 2001, respectively. The average duration of operation was 34 months, with a standard deviation of 2 months. For a comprehensive distribution of the sample across industries, see Figure 1.

### 2.2 Critical incident technique

We employed a modified startup-specific version of the Critical Incident Technique (Flanagan, 1954). In line with Spencer’s competency model (Spencer and Spencer, 1993), we identified core competencies in each narrative that were crucial and whose deficiency played a key role in the startup’s failure. The initial coding for the competencies was based on Spencer and Spencer’s (1993) model. The data were then read to inductively expand the initial codes with startup-specific behavioral descriptions. Through discussions among the coders, startup-specific behavioral descriptions were incorporated into thirteen competencies. Table 1 displays the complete list of competencies, including the startup-specific behavioral descriptions.

**Table 1:** Spencer’s competencies with additional startup-specific behavioral descriptions.

Competency (abbreviation)	Short description by Spencer and Spencer (1993)	Added startup-specific behavior descriptions
Achievement and action cluster		
Achievement orientation (ACH)	Concern for working well or for competing against a standard of excellence	Not applicable (N/A)*
Concern for order, quality, and accuracy (CO)	Reflects an underlying drive to reduce uncertainty in the surrounding environment	Progressing in the right way on startup business development phases. Avoiding perfectionism setbacks. Avoiding superficial and less valid business or technology solutions
Initiative (INT)	A preference for taking action, doing more than is required or expected in the job	N/A*
Information-seeking (INFO)	Making an effort to get more information, not accepting situations at “face value”	Consciously seeking data and information to establish business decisions. Starting and finishing data collection on time. Choosing the right data and information sources and research methods
Helping and human service cluster		
Interpersonal understanding (IU)	Ability to hear accurately and understand the unspoken or partly expressed thoughts, feelings, and concerns of others	Understanding messages from the customer/validation interview
Customer service orientation (CSO)	Focusing efforts on discovering and meeting the customer or client’s needs	Prioritizing customer needs and problems over focus on technological solutions
Competency (abbreviation)	Short description by Spencer and Spencer (1993)	Added startup-specific behavior descriptions
The impact and influence of cluster		
Impact and influence (IMP)	Intention to persuade, convince, influence, or impress others, to get them to support the speakers’ agenda	Influencing potential/actual investors
Organizational awareness (OA)	Ability to understand the power relationships in own organization and the position of the organization in the larger world	N/A*
Relationship building (RB)	To build or maintain friendly, warm relationships or networks of contacts with people who are, or might someday be, useful in achieving work-related goals	Building productive and beneficial relationships with investors and significant / B2B customers
Managerial cluster		
Developing others (DEV)	Intent to teach or to foster the development of one or several other people	N/A*
Directiveness: assertiveness and use of positional power (DIR)	Intent to make others comply with their wishes	Effective and constructive conflict management between founders
Teamwork and cooperation (TW)	Intention to work cooperatively with others, to be part of a team, to work together as opposed to working separately or competitively	N/A**
Team leadership (TL)	Intention to take a role as leader of a team or other group	N/A**
Competency (abbreviation)	Short description by Spencer and Spencer (1993)	Added startup-specific behavior descriptions
Cognitive cluster		
Analytical thinking (AT)	Understanding a situation by breaking it apart into smaller pieces, or tracing the implications of a situation in a step-by-step casual way	Conscious and critical analysis of data and information. Using effective methods and discipline in data analysis
Conceptual thinking (CT)	Understanding a situation or problem by putting the pieces together, seeing the large picture	N/A*
Technical/professional/ managerial expertise (EXP)	Mastery of a body of a job-related knowledge	Including startup-specific knowledge/expertise, e.g., strategic and business planning and management
Personal effectiveness cluster		
Self-control (SCT)	Ability to keep emotions under control and to restrain negative actions when tempted, when working under the conditions of stress	Business decision-making based on facts and data instead of emotional impulses, e.g., avoiding overspending, overstaffing
Self-confidence (SCF)	Belief in own capability to accomplish a task	Business decision-making based on conscious evaluation of own startup’s knowledge, expertise, and activity, e.g., in pricing, contracting
Flexibility (FLX)	Ability to adapt to and work effectively with a variety of situations, individuals, or groups, including change or easily accept changes	Considering experts’ suggestions. Learning from own experiences. Pivoting at the appropriate time, frequency, and direction
Organizational commitment (OC)	Ability and willingness to align own behavior with the needs, priorities, and goals of the organization	Dedicating full working time to own startup when it is required from a business success perspective, avoiding ‘side project’ situations

* did not appear in the sample, ** appeared in the sample, did not get startup-specific additions.

This adapted coding scheme was collaboratively developed by several authors and refined after a trial coding of the data. The data were then independently coded by three trained expert coders (the first three authors of the paper). Their primary task was to identify core competencies within each narrative that, when lacking, played a pivotal role in the startup’s failure. Following manual coding, SPSS (version 24) was used to compute the co-occurrence of deficits.

## 3 Results

### 3.1 Identified competency deficits

A total of 166 instances of competency deficits were identified across the stories, with an average of 3.3 competencies per story. Among the stories, fifteen contained two coded competency deficits. Seventeen leaders mentioned three, eight leaders four, seven leaders five, and three leaders six competency deficits in their testimonials. Of the 166 competency deficits, 37% were coded by all coders, 45% by two coders (with the third coder concurring after discussion), and 18% by one coder (with the other two coders concurring after discussion). These percentages correspond to an average pairwise agreement of 57.8% among the raters, a level of agreement comparable to the interrater agreement in previous studies utilizing the Critical Incident Technique (see Koch et al., 2009 for a discussion on interrater reliability concerning the Critical Incident Technique). Despite the need for discussions to reach a consensus, the coders found the modified Critical Incident Technique effective for identifying competencies in these post-mortem stories.

Two significant competency deficits emerged as failure factors within the sample: a lack of adequate Information-seeking, evident in 35 stories, and unsatisfactory Customer service orientation, appearing in 33 stories. Notably, the CEOs of the startups cited the absence of these competencies as explanations for the eventual failure of their companies in 27 stories, accounting for 54% of the entire sample. Moreover, the absence of Technical expertise emerged as a significant failure factor in 19 stories. Deficiencies in Analytical thinking were attributed to the failure of 18 companies, and Flexibility deficits played a key role in 18 stories. Five competencies -Achievement orientation, Initiative, Conceptual thinking, Organizational awareness, and Developing others- did not emerge as reasons for failure in any of the stories. The frequencies of each competency code are presented in Table 2.

**Table 2:** Competency deficits as reasons for startup failure.

Spencer’s competency deficit	Prevalence in the sample (%)	Example
Achievement and action cluster		
Achievement orientation (ACH)	0	N/A
Concern for order, quality, and accuracy (CO)	12	The team choose a poor platform and ‘quick and dirty’ solutions to build service that led to permanent extra work and loss of traction.
Organizational awareness (OA)	0	N/A
Information-seeking (INFO)	70	The team asked the wrong questions to the wrong people. They received great reviews for the initial product, but did not ask the right people what they would pay for it, and did not generate sufficient revenue.
Helping and human service cluster		
Interpersonal understanding (IU)	12	The team failed to understand the motives of their target group, investors, and therefore could not predict their behavior and willingness in partnering.
Customer service orientation (CSO)	66	The team did not focus on retaining customers, only on new customer acquisition to finance operations and attract investors.
The impact and influence of cluster		
Impact and influence (IMP)	4	The team finally failed to convince the most significant business customers after the product was developed for their needs.
Organizational awareness (OA)	0	N/A
Relationship building (RB)	4	The team did not focus on finding a co-founder and did not choose effective methods to partner with one.
Managerial cluster		
Developing others (DEV)	0	N/A
Directiveness: assertiveness and use of positional power (DIR)	4	The co-founders had significantly different views on important questions, and the minority view was never taken into consideration, leading to poor business decisions.
Teamwork and cooperation (TW)	10	The founders did not deliberately recruit and build the team, therefore key expertise was missing, and they did not find the cooperation effective.
Team leadership (TL)	10	The members of the team had different motives, goals, and interests and the leader failed to align them for the same ‘higher purpose’.
Spencer’s competency deficit	Prevalence in the sample (%)	Example
Cognitive cluster		
Analytical thinking (AT)	36	The team was aware of their goals, investors’ needs, and market specifics, but did not confront these factors, which led to poor decisions.
Conceptual thinking (CT)	0	N/A
Technical/professional/managerial expertise (EXP)	38	The team did not build a comprehensive business model. They only adopted business model elements from other industries that did not work in their case.
Personal effectiveness cluster		
Self-control (SCT)	14	The team moved from individual contractors to employees too early, so the costs skyrocketed.
Self-confidence (SCF)	10	The team was shy to ask a reasonable fee for their service and did not say ‘no’ to customer needs beyond what was paid.
Flexibility (FLX)	36	The team did not consider the mentors’ and other experts’ suggestions and remained with their initial assumptions and ideas.
Organizational commitment (OC)	6	The team had a part-time leader and a part-time developer that significantly limited their traction

*N* = 50.

### 3.2 Co-occurrence of competency deficits

To explore the potential co-occurrence of different competency deficits, we conducted chi-square tests for each possible pair of competencies. In cases where the chi-square test was not applicable due to low cell count, we utilized Fisher’s exact test. A co-occurrence was found between Customer service orientation and Information-seeking *χ*^2^ (1, *N* = 50) = 6.455, *p* = 0.01. Conversely, we observed a significant lack of co-occurrence (i.e., the competency deficits occur separately) between the following pairs of competencies: Information-seeking and Flexibility *χ*^2^ (1, *N* = 50) = 5.357, *p* = 0.02; and Flexibility and Self-control (*p* = 0.04, Fisher’s exact test).

## 4 Discussion

The present study demonstrates a relationship between startup failures and the absence of specific core competencies, as observed from the narrative accounts (post-mortems) provided by CEOs of failed startups. Notably, the most frequently mentioned reasons for failure were deficiencies in Information-seeking and Customer service orientation. This suggests that many startups faced challenges due to limited efforts in consciously seeking and utilizing data and information for decision-making, timely data collection, appropriate data sources, research methodologies, and the insufficient prioritization of customer needs over technological solutions. Our analysis also identified Technical expertise, Analytical thinking, and Flexibility deficits as significant contributing factors to these failures.

Furthermore, although speculative, our findings raise the assumption for future research if Flexibility acted as a mitigating factor against deficiencies in Information-seeking, highlighting the adaptability of the startup unit as crucial in compensating for insufficient attention to external information.

Interestingly, certain core competencies were notably absent from any CEO’s accounts as reasons for failure. These include Achievement orientation, Initiative, Conceptual thinking, Organizational awareness, and Developing others. This observation suggests that startup failure predominantly hinges on stakeholder-related competency deficits in Achievement and action, and Helping and human service clusters, rather than those in the Impact and influence, and Managerial clusters (see Table 2).

These observations align with prior research. Gruber et al. (2008, 2013) emphasized the deficiency in information-seeking and market orientation as key indicators of failure. Other studies have highlighted the importance of industry experience (Maxwell et al., 2011) and team expertise (Carpentier and Suret, 2015). However, our findings, we believe, are not trivial. While our literature review unveils diverse causes for startup failure, factors like development and motivation are frequently mentioned (e.g., Triebel et al., 2018). The lack or low incidence of related competencies (Achievement orientation, Developing others, Initiative, Organizational awareness, Organizational commitment) cast doubt on the prominence of these factors as predictors. It seems that most startups inherently possess these competencies, perhaps due to the specifics of the startup industry (e.g., Achievement orientation, Initiative, and Organizational commitment; as explored by Brandstätter, 2011; Oyeku et al., 2014), or the early stages of organizational formation (Developing others, Organizational awareness; as discussed by Schein, 1983; Gartner, 1985).

### 4.1 Limitations

The sampling technique employed in this research has certain limitations. First, our sole source of information was the CEOs of the startups. Bajwa et al. (2017) suggest that startup CEOs often exhibit a more positive attitude towards failure, verging on “pride in failure”, compared to other types of entrepreneurs and leaders (see also Politis and Gabrielsson, 2009). This predisposition could potentially introduce bias into the recollection and communication of events. Many CEOs of failed startups are serial entrepreneurs (Gruber et al., 2008), and may aim to create a positive image, leading to an overemphasis on stakeholder-related operational competencies that are easier to develop, such as deficient Information-seeking and Customer service, over the absence of more stable self-, team- or organizationally-oriented competencies like Achievement orientation, Conceptual thinking, and Developing others (Salman et al., 2020).

The post-mortems in our study are openly shared and serve as both narrative accounts of failure and components of impression management tactics (Parhankangas and Ehrlich, 2014). This raises questions about the CEOs recollected and interpreted reasons for failure and, consequently, how competency identification might have been influenced by bias (Fischhoff and Beyth, 1975; Tversky and Marsh, 2000).

Secondly, the time intervals between the startup failures and subsequent post-mortem accounts varied across cases, and these narratives were crafted for specific audiences and purposes. Exploring failure stories from diverse perspectives (e.g., investors, customers, co-founders, team members) and employing varied data sources (e.g., business performance metrics) could provide a more comprehensive understanding of the competencies contributing to failure.

Thirdly, while this research blends both qualitative and quantitative approaches, it is important to acknowledge the constraints imposed by the sample size, particularly when dealing with non-salient competencies. Further research in larger and stratified samples might help to refine and validate our findings. However, this is easier said than done, given the limited available data (see, e.g., Shepherd, 2003).

In our study, we examined the co-occurrence of competencies, but not the interactions between team members. According to Pazos et al. (2022) interactions in entrepreneurial teams, namely interpersonal and cognitive conflicts moderate the relationship between individual competencies and team performance, therefore this might be a focus of future studies.

Sevilla-Bernardo et al. (2022) argue in their systematic literature review that the differences in business culture within various environments could potentially influence startup failures. Similarly, the Global Entrepreneurship Monitor (GEM) (2023) reports country-specific variations in entrepreneurial confidence and fear of failure, underscoring the role of cultural factors. It is important to note that our study did not consider these culture-specific variations. Future studies should delve deeper into these culture-related influences, particularly their impact on the success and failure of startups, to fill this gap in the research.

### 4.2 Conclusions and practical applications

Drawing upon the findings, we conclude that Customer service orientation and Information-seeking competencies warrant heightened consideration in the assessment and development of startup teams. The insights from this study have the potential to enhance investor decision-making and mentoring practices. Startup founders and CEOs could greatly benefit from acknowledging the competencies identified as pivotal in failures. This awareness might prompt more calculated progression along the startup journey (Marmer et al., 2012b).

The outcomes of this study can serve as input for nurturing competence development within startup teams – a focus is projected to gain prominence in the forthcoming decade (Prommer et al., 2020).

## Data availability statement

The raw data supporting the conclusions of this article will be made available by the authors, without undue reservation.

## Ethics statement

The studies involving humans were approved by Research Ethics Committee, Faculty of Education and Psychology, Eötvös Loránd University, Budapest, Hungary. The studies were conducted in accordance with the local legislation and institutional requirements. Written informed consent for participation was not required from the participants or the participants’ legal guardians/next of kin in accordance with the national legislation and institutional requirements.

## Author contributions

ES: Conceptualization, Formal analysis, Writing – original draft. ZV: Data curation, Formal analysis, Writing – original draft. AM: Conceptualization, Formal analysis, Writing – review & editing. GN: Conceptualization, Validation, Writing – review & editing. ZS: Validation, Writing – original draft. OK: Supervision, Validation, Writing – review & editing.
